# The *uvrA*, *uvrB *and *uvrC *genes are required for repair of ultraviolet light induced DNA photoproducts in *Halobacterium sp*. NRC-1

**DOI:** 10.1186/1746-1448-2-11

**Published:** 2006-09-13

**Authors:** David J Crowley, Ivan Boubriak, Brian R Berquist, Monika Clark, Emily Richard, Lynn Sullivan, Shiladitya DasSarma, Shirley McCready

**Affiliations:** 1 Natural Sciences Department, Assumption College, 500 Salisbury Street, Worcester, Massachusetts 01609 USA; 2 School of Life Sciences, Oxford Brookes University, Oxford, OX3 0BP, UK; 3 Department of Cell and Developmental Biology, Vanderbilt University, Nashville, Tennessee 37235 USA; 4 University of Maryland Biotechnology Institute Center of Marine Biotechnology Baltimore, Maryland 21042 USA; 5 Greenebaum Cancer Center, University of Maryland, Baltimore, Maryland 21201 USA

## Abstract

**Background:**

Sequenced archaeal genomes contain a variety of bacterial and eukaryotic DNA repair gene homologs, but relatively little is known about how these microorganisms actually perform DNA repair. At least some archaea, including the extreme halophile *Halobacterium sp*. NRC-1, are able to repair ultraviolet light (UV) induced DNA damage in the absence of light-dependent photoreactivation but this 'dark' repair capacity remains largely uncharacterized. *Halobacterium *sp. NRC-1 possesses homologs of the bacterial *uvrA*, *uvrB*, and *uvrC *nucleotide excision repair genes as well as several eukaryotic repair genes and it has been thought that multiple DNA repair pathways may account for the high UV resistance and dark repair capacity of this model halophilic archaeon. We have carried out a functional analysis, measuring repair capability in *uvrA*, *uvrB *and *uvrC *deletion mutants.

**Results:**

Deletion mutants lacking functional *uvrA*, *uvrB *or *uvrC *genes, including a *uvrA uvrC *double mutant, are hypersensitive to UV and are unable to remove cyclobutane pyrimidine dimers or 6–4 photoproducts from their DNA after irradiation with 150 J/m^2 ^of 254 nm UV-C. The UV sensitivity of the *uvr *mutants is greatly attenuated following incubation under visible light, emphasizing that photoreactivation is highly efficient in this organism. Phylogenetic analysis of the *Halobacterium uvr *genes indicates a complex ancestry.

**Conclusion:**

Our results demonstrate that homologs of the bacterial nucleotide excision repair genes *uvrA*, *uvrB*, and *uvrC *are required for the removal of UV damage in the absence of photoreactivating light in *Halobacterium *sp. NRC-1. Deletion of these genes renders cells hypersensitive to UV and abolishes their ability to remove cyclobutane pyrimidine dimers and 6–4 photoproducts in the absence of photoreactivating light. In spite of this inability to repair UV damaged DNA, *uvrA*, *uvrB *and *uvrC *deletion mutants are substantially less UV sensitive than excision repair mutants of *E. coli *or yeast. This may be due to efficient damage tolerance mechanisms such as recombinational lesion bypass, bypass DNA polymerase(s) and the existence of multiple genomes in *Halobacterium*. Phylogenetic analysis provides no clear evidence for lateral transfer of these genes from bacteria to archaea.

## Background

Exposure to the ultraviolet component of sunlight causes DNA damage in cells. After irradiation with 254 nm UV-C, this damage is predominantly cyclobutane pyrimidine dimers (CPDs) and 6–4 photoproducts (6-4PPs) [1,2]. If allowed to persist in the genome, these alterations can cause the blockage of DNA replication and transcription and can lead to the production of point mutations and, ultimately, cell death. Therefore, cells possess a variety of mechanisms that promote survival after UV irradiation, including UV-absorbing pigmentation to protect DNA from damage, repair or removal of the UV photoproducts, cell-cycle checkpoints to prevent premature division in the presence of damage, and damage tolerance mechanisms that allow cells to replicate even when damage remains unrepaired.

A critical repair mechanism for organisms such as plants and aquatic microbes that experience high levels of UV in their natural environment is photoreactivation. This process is dependent on photolyases that absorb and utilize the energy of visible wavelengths of light to reverse the covalent bonds formed between adjacent pyrimidines following UV exposure. Most known photolyases repair CPDs but some repair 6-4PPs [3,4].

Not all organisms possess photolyases but almost all, with the possible exception of some archaea [5,6], have excision repair mechanisms. In bacteria, nucleotide excision repair (NER); i.e. "dark repair" (in contrast to light-dependent photoreactivation) requires the UvrA, UvrB, and UvrC proteins to initiate repair of CPDs and 6-4PPs as well as other bulky lesions; in eukaryotes the NER recognition and incision process involves many more proteins including homologs of *Saccharomyces cerevisiae *RAD1 (XPF in humans), RAD2 (XPG), RAD3 (XPD), RAD4 (XPC), RAD10 (ERCC1), RAD14 (XPA), RAD23 (hhR23a and hhR23b) and RAD25 (XPB). The bacterial and eukaryotic NER systems are operationally similar, but the genes involved are not [7].

Some organisms have an additional alternative excision repair system for UV damage, in which a UV endonuclease (UvsE/Uvde/Uve1) incises immediately 5' to the photoproduct, forming a substrate for a FLAP endonuclease (FEN1/*S. cerevisiae *RAD27) which removes the single-strand DNA 'flap' containing the photoproduct. This latter system is found in organisms as diverse as fission yeast, *Bacillus *species, *Deinococcus radiodurans *and filamentous fungi such as *Neurospora *[6].

Given the variety of repair mechanisms utilized by bacteria and eukaryotes, investigations of DNA repair in archaea are important for understanding the diversity and evolution of repair systems as well as the relationship between these systems and cellular resistance to DNA damage. Although many repair gene homologs – both bacterial and eukaryotic – have been identified in the 27 completely sequenced archaeal genomes, little is known about the functional mechanisms operating in these species [5]. Table 1 shows the NER and photolyase gene homologs that have been identified in archaeal genomes. It appears that there is no universal repair system common to all archaea. Some archaea, all of them euryarchaeota but none of them hyperthermophiles [8], have clear homologs of bacterial NER genes. A few archaea, including *Haloarcula marismortui*, *Haloquadratus walsbyi *and *Methanoculleus marisnigri*, possess genes with homology to the *uvsE*/*uvde *UV-endonuclease genes [9]. Several eukaryotic homologs are also evident in many species. All archaea have homologs of the *S. cerevisiae RAD27 *(human FEN-1) called *rad2*. In addition, most have *RAD3 *and *RAD25 *(human *XPD *and *XPB*) homologs as well as a homolog of *RAD1 *(human XPF), called *eif4a *(Tables 1 &2).

**Table 1:** Homologs of UV repair genes in archaea

		Photoreactivation	Alternative excision repair	Bacterial NER	Eukaryotic NER	Eukaryotic FLAP endonuclease
**Crenarchaeota**	*Sulfolobus tokadaii, S. solfataricus*	*phr*			XPB, XPF	FEN-1
	*Sulfolobus acidocaldarius*	*phr*	*uvsE*		XPB, XPF	FEN-1
	*Pyrobaculum aerophilum*				XPB, XPD, XPF	FEN-1
	*Aeropyrum pernix*				XPB, XPD, XPF	FEN-1
**Euryarchaeota**	***Halobacterium sp. NRC-1***	***phr1, phr2***		***uvrA, uvrB, uvrC, uvrD***	**XPB, XPD, XPF**	**FEN-1**
	*Thermoplasma volcanium, T. acidophilum*				XPB, XPD,	FEN-1
	*Methanothermobacter thermoautotrophicus*	*phr*		*uvrA, uvrB, uvrC*	XPF	FEN-1
	*Archaeoglobus fulgidus*				XPB, XPD, XPF	FEN-1
	*Haloarcula marismortui*	*phr1, phr2*	*uvsE*	*uvrA, uvrB, uvrC, uvrD*	XPB, XPD, XPF	FEN-1
	*Pyrococcus horikoshii, P. abyssi, P. furiosus*				XPB, XPD, XPF	FEN-1
	*Methanopyrus kandleri*	*phr*			XPB, XPF	FEN-1
	*Methanococcus jannaschii*				XPB, XPF	FEN-1
	*Methanosarcina mazei, M. acetivorans, M. barkeri*	*phr*		*uvrA, uvrB, uvrC, uvrD*	XPB, XPD, XPF	FEN-1

**Table 2:** Selected gene homologies relevant to this work

*Halobacterium*	Bacteria	*S. cerevisiae*	*S. pombe*	human	Function
*rad2*^1^		*RAD27*^1^	*rad2*^1^	FEN-1^1^	structure-specific endunuclease makes 3' incision in base excision repair
*rad3a, rad3b*		*RAD3*	*rad15*	XPD	5' to 3' helicase
*rad25a, rad25b*		*RAD25*		XPB	3' to 5' helicase
		*RAD2*	*rad13*	XPG	makes 3' incision in eukaryotic NER
*eif4a*		*RAD1*	*rad16*	XPF	makes 5' incision in eukaryotic NER (with *RAD10*/ERCC1)
*uvrA, uvrB, uvrC, uvrD*	*uvrA, uvrB, uvrC, uvrD*				recognition, incision and removal of damaged DNA in bacterial NER

Notes

^1 ^*S. cerevisiae RAD2*/human XPG belongs to the same multigene family as *S. cerevisiae RAD27*/FEN-1 but the *Halobacterium *sp. NRC-1homolog, *rad2*, is more closely related to *RAD27*/FEN-1. There is no other homolog in *Halobacterium *sp. NRC-1.

It has been speculated [10] that archaea may employ a simplified form of eukaryotic NER, in which Eif4a and Rad2 make incisions on either side of the lesion before a Rad3/Rad25 helicase removes the damaged region. Although archaea lack the XPA and XPC homologs involved in preincision steps of eukaryotic NER, it was suggested that they may be dispensable in this putative, stripped-down 'ancestral' form of NER. The observed UV repair patch size of 10–11 bp in *Methanobacterium thermoautotrophicum *[11] and the presence of *uvrA*, *uvrB *and *uvrC *genes in this organism, suggests a bacterial form of NER.

*Halobacterium *sp. NRC-1 has been useful as a model system to examine the response of archaea to environmental stressors like UV [12,13]. The organism is adapted to extremely halophilic brine, such as in the Great Salt Lake and marine salterns, which are used to mine salt from the sea [14]. The organism is exposed to intense solar radiation in these environments and has a high tolerance to UV irradiation [12,15,16] The sequenced genome of *Halobacterium *sp. NRC-1 reveals both bacterial and eukaryotic repair gene homologs and therefore it has been proposed that this organism may employ multiple DNA repair mechanisms to thrive in its natural habitat [17,18]. We have initiated a functional analysis of putative UV repair pathways by constructing deletion mutants lacking the bacterial NER homologs *uvrA*, *uvrB *and/or *uvrC*. We have measured their UV sensitivity and their ability to repair UV damage to determine whether the Uvr proteins operate in a functional repair pathway and, if they do, whether there is residual repair capacity in their absence, which would indicate an additional repair pathway(s).

## Results

### Construction of *uvr *mutants

Genetic knockouts of each *Halobacterium *sp. NRC-1 *uvr *gene homolog were constructed to determine whether these genes are functionally homologous to the bacterial *uvrA*, *uvrB*, and *uvrC *genes that are required for NER in *Escherichia coli *and other bacteria [19]. Knockouts of the *Halobacterium sp*. NRC-1*uvr *genes were constructed using an established *ura3 *counterselection strategy in which a *ura3*^+ ^non-replicating *Halobacterium *shuttle vector (pMPK428) was engineered to carry a deletion construct composed of two 400–500 bp fragments that flank the region to be deleted [20,21]. These constructs were engineered for the *uvrA*, *uvrB*, and *uvrC *sequence homologs from *Halobacterium *sp. NRC-1 and the resulting plasmids were named pDC*ΔuvrA*, pDC*ΔuvrB*, and pDC*ΔuvrC*. Each plasmid was transformed into DJC501, a *ura3*^- ^derivative of *Halobacterium sp*. NRC-1. *ura3*^+ ^primary integrants were selected by plating on a medium deficient in uracil and, following PCR-based confirmation of plasmid integration, these primary integrants were cultured in a uracil-deficient medium and subsequently plated on rich medium containing 5-Fluoroorotic acid, which is toxic to *ura3 *prototrophs. Only cells which lost the deletion plasmid through a second recombination event and restored the *ura3*^- ^genotype could form colonies on 5-FOA plates. 5-FOA-resistant colonies were cultured and screened by PCR for the presence of deletion alleles.

**Figure 1:**
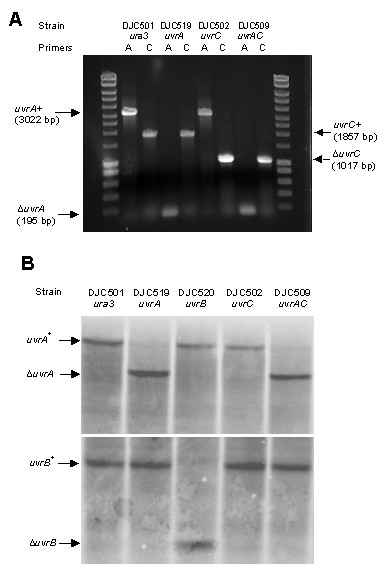
**PCR and Southern hybridization to confirm deletion genotypes**. PCR and Southern hybridizations were performed on genomic DNA isolated from the indicated strains. (**A**) For *uvrA*, PCR will amplify a 3022 bp fragment if the wild-type allele is present and a 195 bp fragment if the deletion allele is present. For *uvrC*, presence of the wild-type allele is indicated by amplification of an 1857 bp fragment, the deletion allele by amplification of a 1017 bp fragment. Lanes 1 and 10 contain Hyperladder I for reference (BioLine). PCR primers are listed in Table 3. Similar results were obtained using primers for *uvrB *(data not shown). (**B**) Genomic DNA was digested with *Pst*I and, following electrophoresis and transfer to a charged membrane, was hybridized with chemifluorescently labelled PCR fragments to a region upstream of the *uvrA *gene (top) or *uvrB *gene (bottom). Similar results were obtained for blots hybridized with probes to the *uvrC *genomic region (data not shown).

Using the appropriate primer sets for PCR (Table 3), results such as those presented in Figure 1 were obtained. The control *ura3*^- ^strain, DJC501, contained wild-type alleles of *uvrA *and *uvrC *(Figure 1A, lanes 2 and 3). In contrast, the strains DJC519 and DJC502 contained the deletion alleles of *uvrA *and *uvrC*, respectively (Figure 1A, lanes 4 and 7). As expected, both strains possessed wild-type alleles of the non-targeted gene (Figure 1A, lanes 5 and 6). In the double mutant DJC509, both *uvrA *and *uvrC *deletion alleles were amplified (Figure 1A, lanes 8 and 9). Similar results were obtained for strain DJC520 (*uvrB*, data not shown). Because *Halobacterium *sp. NRC-1 is reputed to carry up to 30 copies of the genome (J. Soppa, personal communication), it was important to show not only the presence of the deletion allele but also the absence of any wild-type copies in the putative mutant strains. To do this, we carried out Southern hybridizations for each *uvr *gene. The Southerns confirmed that no *uvr*^+ ^alleles were present in any of the mutant strains (Figure 1B and data not shown).

**Table 3:** PCR primers Primers used in PCR for constructing deletions and for screening *uvr *alleles. All listed 5'→3'. Engineered restriction sites underlined

**Primers for constructing deletions:**	
uvrA1 forward	GGGGGTACCGTATTTGTTCGGCACGAGGT
uvrA1 reverse	GGGTCTAGACTCTTCGCTCATTGGGAGAG
	
uvrA2 forward	GGGTCTAGACTCTCGCGGCTCTGTCTC
uvrA2 reverse	GGGAAGCTTACCGTCTCAGTGGTGGTGTC
	
uvrB1 forward	GGGGGTACCAGGAACGCGACCACTACG
uvrB1 reverse	GGGTCTAGAGCTGGCGTCACTCATTACAC
	
uvrB2 forward	GGGTCTAGAGAAACCGAGGACTGGTGAGA
uvrB2 reverse	GGGAAGCTTGGGAACACGAAGATGAGGAA
	
uvrC1 forward	GGGGGTACCGTACGTGGGTGTGATGAACG
uvrC1 reverse	GGGTCTAGATTCACGACTGTCTCCACGTC
	
uvrC2 forward	GGGTCTAGAAGAACGACGACTACGCGAAC
uvrC2 reverse	GGGAAGCTTACGTCTCGGAGTACCAGCAG
	
**Primers for screening deletions by PCR**	
*uvrA *up	AATGTCGTAGTCGGCCATGT
*uvrA *down	CACAGCCCCGAGACAGAG
	
*uvrB *up	GGCCTACGACGAGTACACC
*uvrB *down	TGAAAAGCGTTGGTTTCTCC
	
*ura3 *up	CTTCCGGAGGACGTACAGG
*ura3 *down	CGTACTGGGCGTTCCACT
	
*bla *up	TTTGCCTTCCTGTTTTTGCT
*bla *down	TTGCCGGGAAGCTAGAGTAA

### *uvrA*, *uvrB*, *uvrC*, *and uvrAuvrC *mutants are UV sensitive in the dark

**Figure 2:**
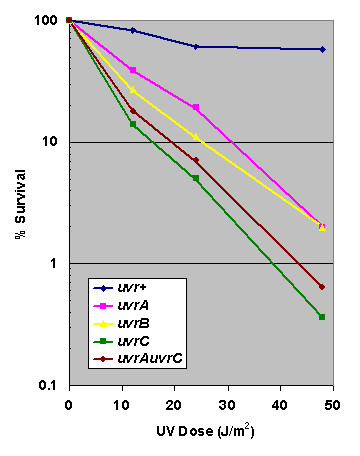
***Halobacterium uvrA, uvrB, uvrC, and uvrAuvrC mutants *are sensitive to UV light**. Data represent the averages of at least three independent survival experiments. Standard deviations between all *uvr *mutants and DJC501 (*uvr*^+^) were non-overlapping. All manipulations and incubations were performed in yellow light or in the dark to prevent photoreactivation.

If the *uvr *genes are necessary for dark repair of UV-induced photoproducts in *Halobacterium*, we predicted that our deletion strains would be hypersensitive to UV when irradiated and incubated in non-photoreactivating conditions. We performed quantitative survival assays on DJC501 and the *uvr *deletion mutants we constructed and found a high degree of UV sensitivity, in the dark, in strains carrying mutant alleles of *uvrA*, *uvrB *or *uvrC *(Figure 2). In these mutant strains, approximately two logs of cell killing were observed at a dose (48 J/m^2^) resulting in well over 50% survival in the control DJC501 *uvr*^+ ^strain.

Restoration of the *ura3*^- ^genotype in the counterselection knockout strategy allows for multiple deletions to be made in a single strain. If the *uvr *genes are performing a coordinated excision repair process similar to that of bacteria, then deletion of a second gene in this pathway should not further sensitize the cells to UV. To test this, DJC 502 (*uvrC*) was transformed with pDC*ΔuvrA *to construct DJC 509 which carries deletions of both the *uvrA *and the *uvrC *genes (Figure 1). As can be observed in Figure 2, this double mutant was no more sensitive to UV than either the *uvrA *or *uvrC *single mutants, confirming that these genes operate in the same pathway in *Halobacterium *sp. NRC-1, presumably nucleotide excision repair.

**Figure 3:**
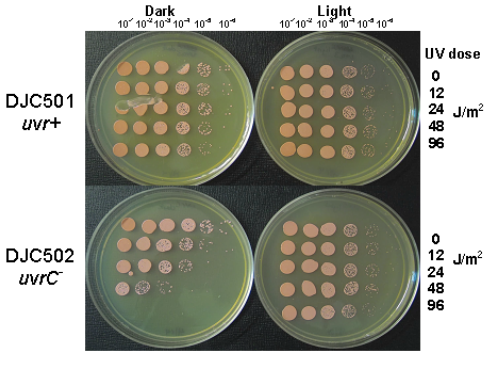
**Photoreactivation restores survival of *uvr *mutants after UV**. To observe the effects of photoreactivation on survival of DJC501 (top panel, *uvr*^+^) and DJC502 (bottom panel, *uvrC*), cells from each strain were UV irradiated at the doses shown, subjected to 10-fold dilution series and identically spotted on two plates. The spotted plates were exposed to fluorescent light for 24 hours prior to incubation for 4–5 days at 42°C. The plates on the left (''Dark'') were wrapped in aluminum foil during the fluorescent exposure while those on the right (''Light'') were left unwrapped.

Previous studies have shown that photoreactivation is highly efficient in the halophilic archaea, including *Halobacterium *sp. NRC-1, which encodes the CPD photolyase *phr2 *[16,22]. In some bacteria and eukaryotes, molecular interactions between photoreactivation and excision repair have been suggested [23]. To test the effect, if any, of the *uvr *genes on photoreactivation, we performed experiments in which identical plates inoculated with UV-treated *uvr*^+ ^or *uvr *mutant cells were exposed to photoreactivating conditions (24 hours of fluorescent bulb irradiation) or kept wrapped in aluminum foil. For all strains, minimal loss of viability was detected on the unwrapped plates after 48 J/m^2 ^UV, indicating efficient photoreactivation that was not dependent on *uvr *genes (Figure 3 and data not shown).

### Uvr mutants are completely deficient in dark repair

**Figure 4:**
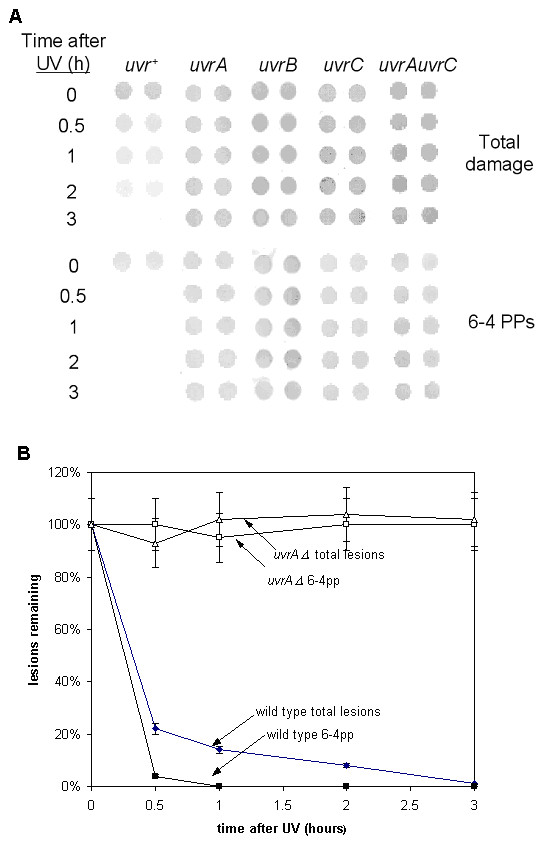
***Halobacterium uvrA, uvrB, uvrC and uvrAuvrC mutants *are completely deficient in dark repair of UV-induced photoproducts**. (**A**) Sample dot blots (in duplicate) of total damage (CPDs and 6–4PPs, top) and 6-4PPs alone (bottom) for DNA samples isolated at the indicated times following 150 J/m^2 ^UV-C treatment to DJC501 (*uvr*^+^), DJC519 (*uvrA*), DJC520 (uvrB), DJC502 (*uvrC*) and DJC509 (*uvrAuvrC*). (**B**) Total damage and 6-4PPs from *uvr*^+ ^and *uvrA *mutant strains during 3 hours post-UV incubation, showing repair in the wild-type and no repair in the mutant.

Given the sensitivity of the *uvr *mutants to UV and their predicted function in excision repair of UV damage, we measured the ability of the deletion mutants to remove CPDs and 6-4PPs after a dose of 150 J/m^2 ^UV-C. The data show that the *uvr*^+ ^cells repaired virtually all the 6-4PPs and around 80% of CPDs within one hour, and almost all damage in 3 hours, but that the *uvrA*, *uvrB*, and *uvrC *single mutants as well as the *uvrA uvrC *double mutant were unable to repair any CPDs or 6-4PPs during 3 hours post-irradiation incubation (Figure 4). This confirms that these genes are absolutely required for repair of UV lesions in the absence of photoreactivation, indicating that there is no other 'dark' repair mechanism in *Halobacterium sp*. NRC-1. This does not preclude the possibility that some of the eukaryotic repair homologs such as *rad3 *or *rad25 *are also involved, though NER proceeds without the need for these proteins in bacteria.

### Phylogenetic analysis of bacterial and archaeal Uvr proteins

**Figure 5:**
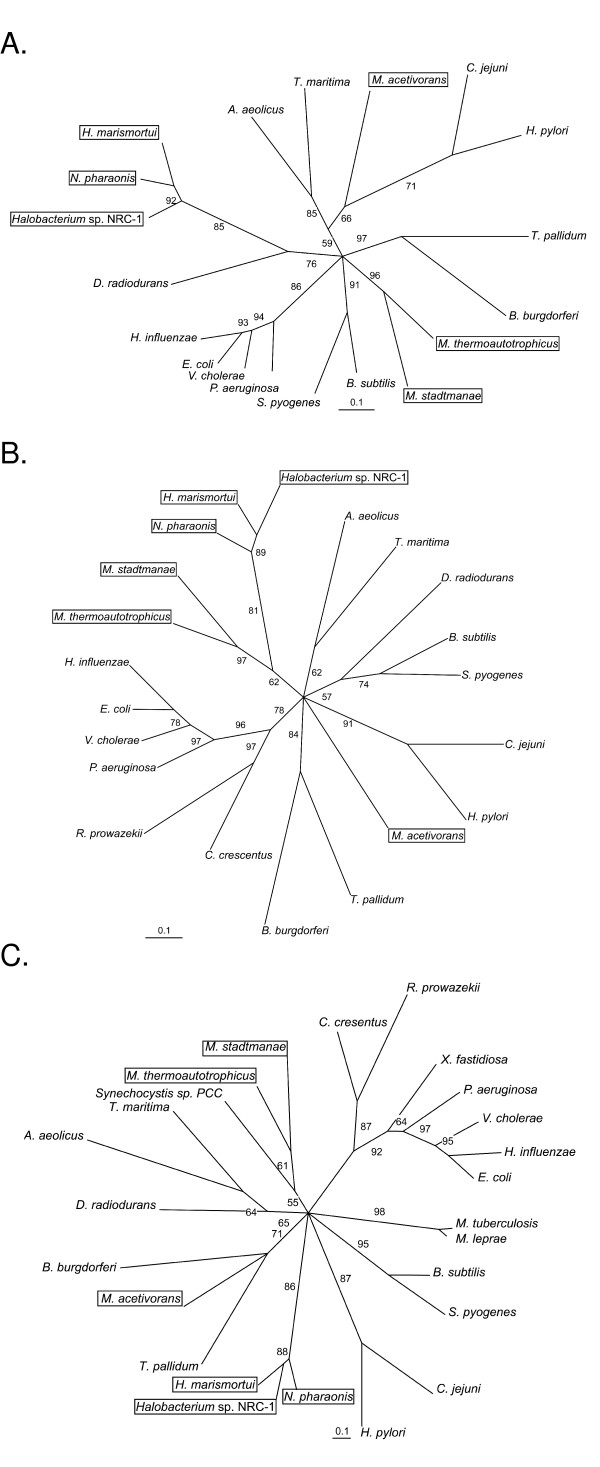
**Quartet puzzling consensus maximum likelihood phylogenetic analysis of Uvr proteins encoded in archaeal genomes and representative bacteria**. Phylogenetic analysis of UvrA (A), UvrB (B) and UvrC (C) protein sequences from haloarchaea, mesophilic methanogenic archaea, and representative bacteria.

The *uvrA*, *uvrB*, and *uvrC *genes are found in the halophilic archaea and mesophilic methanogenic archaea but are absent from the genome sequences of all other archaea sequenced to date. Given this distribution, we examined the phylogenetic relationships between the core archaeal-encoded proteins (for all archaea known to contain them) and protein sequences found in a few diverse families of bacteria. Phylogenetic analysis of each of the Uvr sequences gave star topologies at the root, indicating that origins of the protein sequences cannot be uncovered. Haloarchaeal UvrA, UvrB, and UvrC always formed a monophyletic clade. Sequences from the mesophilic methanogenic archaea, however, were paraphyletic, with sequences from *Methanosarcina acetivorans *being quite different from the sequences encoded in the genomes of *Methanothermobacter thermoautotrophicus *and *Methanosphaera stadtmanae*. For UvrA, the haloarchaea were found to group with the UvrA sequence from the extremely radiation resistant bacterium *Deinococcus radiodurans*, while *M. thermoautotrophicum *and *M. stadtmanae *formed their own unique clade. *M. acetivorans *UvrA branched with the enterobacteria *Camphylobacter jejuni *and *Helicobacter pylori *(Figure 5A). For UvrB, the haloarchaea, *M. thermoautotrophicum*, and *M. stadtmanae *formed a major monophyletic clade together, while UvrB from *M. acetivorans *branched off on its own (Figure 5B). For UvrC, the haloarchaea formed a unique monophyletic clade, *M. thermoautotrophicum *and *M. stadtmanae *formed a clade with the cyanobacterium *Synechocystis *sp. PCC, and *M. acetivorans *claded with the spirochetes *Borrelia burgdorferi *and *Treponema pallidum *(Figure 5C).

## Discussion

Our data clearly demonstrate the functional homology of the *Halobacterium *sp. NRC-1 *uvrA*, *uvrB*, and *uvrC *genes to the same genes found in bacteria. In the absence of any one of these genes, cells are significantly more sensitive to UV light (Figure 2) and removal of UV-induced photoproducts is effectively abolished in the absence of photoreactivating light (Figure 4). Moreover, cells deficient in both *uvrA *and *uvrC *showed no enhanced sensitivity, indicating that these genes operate in the same pathway in *Halobacterium*. We conclude the *uvrA*, *uvrB*, and *uvrC *genes encode proteins that perform NER of UV photoproducts and that this pathway is required for the removal of these lesions in *Halobacterium *in the absence of photoreactivation.

The extremely halophilic archaea with published genome sequences (*Halobacterium *sp. NRC-1, *Haloarcula marismortui *and *Natronomonas pharaonis*) all carry the bacterial-type *uvr *genes so it seems likely that they all perform functional bacterial-type NER. *Haloferax volcanii *has been shown to excise UV lesions [15], but its complete genome sequence is not yet available. The bacterial *uvr *homologs found in the genome of the mesophilic methanogen *Methanobacterium thermoautotrophicum *seem to be functional as well, since a repair patch size of 10–11 bases has been measured, which is typical of the bacterial NER patch size [11].

Many archaea do not possess homologs of the bacterial-type *uvrABC *genes, however, and it is likely that they employ alternative excision repair mechanisms to remove bulky lesions from DNA. 'Dark' repair of CPDs in *Sulfolobus solfataricus *(which does not have the bacterial-type *uvr *genes, Table 1) after a dose of 200 J/m^2 ^has been reported [24]. It was suggested that this repair involves the eukaryal NER genes, *RAD1*/XPF/ERCC4, *RAD2*/XPG and *RAD25*/XPB (which were reported to be up-regulated by UV) and *RAD3*/XPD [24].

In contrast, our data suggest that the eukaryotic repair homologs present in the *Halobacterium *genome are not involved in NER. The eukaryotic RAD proteins have roles in a variety of biochemical pathways besides repair, including DNA replication (*RAD2*, [25]), recombinational repair (*RAD1*, [26,27]), and transcription (*RAD2, RAD3, RAD25*, [28,29]). It may be that the archaeal homologs of these genes are also involved in these or other non-NER pathways in *Halobacterium*. However, it is possible that the *Halobacterium rad *genes, and perhaps others in the genome, are involved in promoting sub-pathways of NER, particularly transcription-coupled repair (TCR), which requires the coupling of NER to an RNA polymerase arrested at DNA lesions. This sub-pathway of repair has yet to be demonstrated in the archaea, but has been observed in a wide range of bacteria and eukaryotes [30]. Homologs of the *rad3 *and *rad25 *putative DNA helicases are required for TCR in yeast [31] and may operate in this capacity in *Halobacterium*. These genes may also operate in a primary role following damage incision by UvrABC proteins, or in a very efficient back-up role to the predicted NER helicase, UvrD.

In its natural sunlit habitat, *Halobacterium *performs efficient photoreactivation to repair UV-induced photoproducts [16,22,32]. In our *uvr *mutants, photoreactivation remained highly effective, supporting complete survival after 48 J/m^2 ^UV when cells were exposed to fluorescent light after UV (Figure 3). Given the remarkable efficiency of photoreactivation, we must question the role that the *uvr*^-^dependent repair system plays in this organism. In most organisms, photoreactivation is directed towards CPDs, the major UV-induced lesion, and NER is solely responsible for repair of 6-4PPs. However, 6-4PPs also appear to be a target of photoreactivation in *Halobacterium *although the mechanism for this direct repair has not yet been elucidated [16]. In the absence of a primary role in repairing UV lesions, perhaps a major role of the *uvr*-dependent repair system in *Halobacterium *is to monitor the genome for a variety of other bulky DNA lesions. The *uvr *genes may also be targeted to lesions in expressed genes through transcription-coupled repair. In this way, transcription-arresting lesions would be targeted for removal by *uvr*-based NER, promoting gene expression after DNA damage. The *E. coli *UvrABC proteins are absolutely required for TCR and are coupled to an arrested RNA polymerase by the Mfd protein [33]. No Mfd orthologs have been found in *Halobacterium*, which is not surprising given that the archaeal RNA polymerase is much more similar to the eukaryotic RNA polymerase II complex than the bacterial transcription machinery. It will be important to determine whether transcription-coupled repair occurs in *Halobacterium *and if the *uvr *and *rad *genes are involved in this process.

Compared to similar NER knockouts in other organisms [19,34]*Halobacterium uvr *mutants are relatively resistant to UV (Figure 2). Given the lack of UV protective mechanisms afforded by membrane pigments ([15,35] McCready & Crowley, unpublished observations) and the absence of any detectable repair of UV lesions in these mutants, it appears that these organisms possess proficient UV damage tolerance mechanisms. These mechanisms may include damage-inducible mechanisms involving the protein RadA1. The *radA1 *gene is highly induced (7-fold) after UV, suggesting that it plays a critical role in tolerance of DNA damage [12] and it has been suggested that RadA1 may participate in rescuing stalled or collapsed replication forks, allowing (error-free) lesion bypass in the absence of repair [36,37]. In addition, *Halobacterium *sp. NRC-1 encodes at least one lesion bypass polymerase belonging to the DinB/UmuC/Rad30/Rev1 DNA polymerase superfamily [38], which facilitate bypass of photoproducts and allow replication to continue on damaged templates. Another important factor is likely to be the presence of multiple copies of the genome in *Halobacterium *cells. Up to 30 copies per cell have been observed (Soppa, personal communication; [39]) which could conceivably permit damaged cells to survive and reproduce, through segregation of undamaged chromosomes, perhaps facilitated by RadA1-mediated recombinational mechanisms.

Our phylogenetic analysis shows a complex history for the Uvr proteins in archaea. Although it is tempting to speculate that the genes encoding these proteins were laterally transferred into the different archaea from bacteria, it is also possible that the Uvr system was present in an ancestral archaeon and subsequently lost from most extant lineages identified thus far. If lateral transfer of these genes did occur, the system was probably put together in a piecemeal fashion, with acquisition of individual genes in archaea coming from diverse bacterial groups.

## Conclusion

We conclude that the bacterial-type *uvrA*, *uvrB *and *uvrC *genes are absolutely required for repair of UV photoproducts in *Halobacterium *sp. NRC-1 and that this pathway is solely responsible for excision repair of UV lesions from the genome of this archaeon.

## Methods

### Construction of *uvr *deletion mutants

All mutants were constructed using published techniques [20,21]. In brief, 400–500 base pair (bp) flanking regions of each *uvr *gene were amplified by PCR and cloned into pMPK428 (generous gift of M. Krebs and R. Peck), which carries the wild-type allele of *ura3 *and the β-lactamase (*bla*) gene for ampicillin selection in *E. coli*. PCR primers targeted to the upstream flanking region of each gene were engineered with *Kpn*I and X*ba*I sites on the forward and reverse primers, respectively (see Table 3). Primers targeted to the downstream region of each gene were similarly engineered with *Xba*I and *Hin*dIII sites. Following amplification and purification, the PCR fragments were digested with the appropriate enzymes and triple-ligated with pMPK428 digested with *Kpn*I and *Hin*dIII. The ligation mixtures were transformed into competent *E. coli *(JM109) cells and transformants were selected by plating on LB agar containing100 μg/ml ampicillin. Ampicillin-resistant colonies were picked, cultured, and plasmids were purified and digested with *Hin*dIII and *Kpn*I to check for appropriate inserts. Selected plasmids with predicted restriction patterns were sequenced and named pDC*ΔuvrA*, pDC*ΔuvrB*, and pDC*ΔuvrC*.

*Halobacterium sp*. NRC-1 *ura3*^- ^cells (a generous gift of Dr. M. P. Krebs, renamed DJC501) were grown to mid-log phase in modified rich CM+ media (per liter: 250 g NaCl, 20 g MgSO_4_·7H_2_O, 3 g Na citrate, 2 g KCl, 5 g Bacto-Tryptone, 3 g yeast extract, 1 g casamino acids pH 7.2, plus trace metals) and transformed with pDC*ΔuvrA*, pDC*ΔuvrB*, and pDC*ΔuvrC *in separate reactions following established techniques [14,40]. Primary integrants (via homologous regions in deletion construct) were selected by plating transformation mixtures on HURA plates (per liter: 250 g NaCl, 20 g MgSO_4_·7H_2_O, 3 g Na citrate, 2 g KCl, 10 g of nitrogen base (Sigma-Aldrich co. Y0626), 1.92 g synthetic uracil dropout formula (Sigma-Aldrich co. Y1501), 20 g agar, pH 7.0 [21] on which only *ura3*^+ ^cells can grow. Primary integrant colonies were picked, grown to log phase in HURA broth, and DNA was prepared. PCR was performed using primers for the *ura3 *and *bla *genes to confirm integration of deletion plasmids (see Table 3). Log-phase cultures of *ura*^+^*bla*^+ ^primary integrants were plated on modified CM+ plates (as above with 20 g agar/liter) + 0.25 mg/ml 5-fluoroorotic acid (5-FOA; Research Products International Corp., F10501) to select for loss of plasmid (and its *ura3*^+ ^allele) via a second homologous recombination event. In approximately 50% of 5-FOA resistant colonies, the plasmid was lost by a recombination event that resulted in replacement of the targeted wild-type allele with the engineered deletion construct. 5-FOA resistant colonies were screened by PCR and genotypes confirmed by Southern blotting and hybridization.

### Screening of putative deletion mutants

5-FOA resistant colonies were cultured at 42°C in a C76 water bath shaker (New Brunswick Scientific, Edison, N.J.) at 200 rpm in modified rich CM+ liquid media and genomic DNA was prepared as described [40]. PCR was performed using primer sets shown in the bottom section of Table 3. For *uvrC *amplifications, the uvrC1 forward and uvrC2 reverse primers were used.

Genomic DNA was digested with *Pst*I (*uvrA *and *uvrB*), or *Kpn*I and *Pvu*II (*uvrC*) for determining genotype by Southern blot hybridization. Samples were subjected to electrophoresis overnight in alkaline gels containing 0.8% agarose. DNA was transferred to Hybond N+ membranes by Southern blotting and hybridized with AlkPhos-Direct with ECF-labelled (GE Healthcare Life Sciences, RPN3692) PCR fragments from either the upstream or downstream flanking region of each gene (Table 3). Chemifluorescence signal was detected using a GE Healthcare Storm Imager.

### Quantitative UV survival curves

*Halobacterium *cultures were grown to mid-log phase in modified CM+ liquid media, centrifuged, and washed twice with CM salts (per liter: 250 g NaCl, 20 g MgSO_4_·7H2O, 3 g Na citrate, 2 g KCl, pH 7.2). Working in subdued yellow light to prevent photoreactivation, washed cells were irradiated with a 254 nm germicidal lamp at a dose rate of 0.8 J/m^2^/sec with mild agitation. Samples were diluted and 20 microliters of each dilution were spotted on modified CM+ plates. Plates were wrapped in foil and incubated 4–5 days at 42°C. To observe the effects of photoreactivation on survival, two identically spotted plates were exposed to Sylvania Gro-Lux fluorescent light (Sylvania F40/GRO/AQ/RP) for 24 hours prior to incubation at 42°C. One plate was wrapped in foil as a control.

### Repair experiments and Immunoassays for measurement of photoproducts

The repair experiments and dot-blot immunoassays for UV photoproducts were carried out as described previously [12,15,21,41]. Log-phase cells were harvested and irradiated with 150 J/m^2 ^UV-C at a dose rate of 1 J/m^2^/sec and the irradiated cells were incubated aerobically at 37°C to allow repair to proceed. We have previously shown that, after this dose of UV, there is no detectable DNA replication during a 3-hour post-UV incubation [15]. All irradiation and post-UV incubation was carried out either under yellow light illumination or in the dark. Fifty-ml samples were harvested at timed intervals and genomic DNA extracted using Promega Wizard genomic DNA kits. DNA samples from the various time points were equalised by measuring fluorescence of ethidium bromide-stained DNA in agarose gels, adjusting DNA concentrations and repeating this analysis as many times as necessary until all samples were of equal concentration.

Two identical dot blots were prepared on nitrocellulose filters, each containing a set of dilutions of each DNA sample in 1 M ammonium acetate. One blot was used to measure total damage (CPDs and 6-4PPs); the other was exposed to CPD photolyase and visible light to eliminate CPDs and allow for detection of 6-4PPs alone. The blots were then exposed to rabbit polyclonal antiserum containing antibodies to 6-4PPs and CPDs, then to biotinylated anti-rabbit antibody followed by alkaline phosphatase-conjugated Extravidin (Sigma) and finally to Nitro Blue tetrazolium (NBT) and 5-bromo-4-chloro-indolyl phosphate (BCIP) substrate. The intensity of blue color was proportional to the amount of DNA damage in the samples and was measured using a scanning densitometer (BioRad GS-670) and compared to a set of standards included on each blot.

Phylogenetic analysis of Uvr protein sequences in archaeal genomes

Protein sequences for the core Uvr system components (UvrA, UvrB, and UvrC) from representative bacteria, *Halobacterium *sp. NRC-1 and *Methanothermobacter thermoautotrophicus*, were downloaded from COGs 0178, 0556 and 0322, respectively. Sequences for *Haloarcula marismortui, Natronomonas pharaonis*, and *Methanosphaera stadtmanae *were downloaded from NCBI (see Additional file 1). Amino acid sequences were aligned using CLUSTAL_X1.83 [42]. Alignments were manually inspected and edited if necessary. TREEPUZZLE5.2 was used for quartet puzzling consensus maximum likelihood phylogenetic reconstruction using the JTT amino acid substitution matrix [43].

## Competing interests

The author(s) declare that they have no competing interests.

## Authors' contributions

DJC and SM conceived and designed the study and co-wrote the manuscript. DJC and MC constructed the mutants and carried out preliminary characterization. ER, LS and IB confirmed mutant genotypes by PCR and Southern analysis; ER and LS performed UV survival experiments. IB carried out the repair experiments; SM carried out the immunoassays. BB and SD performed the phylogenetic analysis and assisted with preparation of the manuscript. All authors read and approved the final manuscript.

## Supplementary Material

Additional File 1 Accession numbers for phylogenetic analysis. Accession numbers used to generate data presented in Figure 5. Click here for file
