# Supplementary material for: The uvrA, uvrB and uvrC genes are required for repair of ultraviolet light induced DNA photoproducts in Halobacterium sp. NRC-1
Source: Saline Syst. 2006 Sep 13;2:11. doi: 10.1186/1746-1448-2-11 (PMC1590041; doi:10.1186/1746-1448-2-11)
Supplement: Additional File 1 — Accession numbers for phylogenetic analysis. Accession numbers used to generate data presented in Figure 5. [file 1746-1448-2-11-S1.doc]

Additional File 1 - Accession numbers for phylogenetic analysis

| Species | UvrA | UvrB | UvrC |
| --- | --- | --- | --- |
| *Halobacterium* NRC-1 | NP_281002 | NP_281002 | NP_280996 |
| *N. pharaonis* | YP_326032 | YP_326032 | YP_326203 |
| *H. marismortui* | YP_137431 | YP_137431 | YP_137470 |
| *M. acetivorans* | NP_618212 | NP_618212 | NP_618213 |
| *M. thermoautotrophicus* | NP_275586 | NP_275585 | NP_275584 |
| *M. stadtmanae* | YP_447615 | YP_447615 | YP_447614 |
| *D. radiodurans* | NP_295996 | NP_295996 | NP_295077 |
| *A. aeolicus* | NP_214276 | NP_214276 | NP_214456 |
| *T. maritima* | NP_229558 | NP_229558 | NP_228078 |
| *C. jejuni* | NP_281852 | NP_281852 | NP_282393 |
| *H. pylori* | NP_207905 | NP_207905 | NP_207614 |
| *T. pallidum* | NP_207905 | NP_218556 | NP_218913 |
| *B. burgdorferi* | NP_212970 | NP_212970 | NP_212591 |
| *B. subtilis* | NP_391397 | NP_391397 | NP_390727 |
| *S. pyogenes* | NP_219436 | NP_219436 | NP_269234 |
| *C. cresentus* | NP_421775 | NP_421775 | NP_421677 |
| *R. prowazekii* | NP_220591 | NP_220591 | NP_220944 |
| *E. coli* | NP_415300 | NP_415300 | NP_416423 |
| *H. influenzae* | NP_439403 | NP_439403 | NP_438230 |
| *V. cholerae* | NP_230664 | NP_230664 | NP_230859 |
| *P. aeruginosa* | NP_251828 | NP_251828 | NP_251275 |
